# Supplementary material for: Peculiar Morphologies Obtained for 80/20 PLA/PA11 Blend with Small Amounts of Fumed Silica
Source: Nanomaterials (Basel). 2021 Jun 29;11(7):1721. doi: 10.3390/nano11071721 (PMC8308147; doi:10.3390/nano11071721)
Supplement: Supplementary file 1 [file nanomaterials-11-01721-s001.zip › nanomaterials-1254545-supplementary.pdf]

nanomaterials

Supplementary Materials

Peculiar Morphologies Obtained for 80/20 PLA/PA11 Blend with Small Amounts of Fumed Silica

Damien Rasselet [1], Monica Pucci [2], Anne-Sophie Caro-Bretelle [2], José-Marie Lopez-Cuesta [1] and Aurélie Taguet [1,*]

[1] Polymers Composites and Hybrids (PCH), IMT Mines Ales, 30319 Ales, France; damien.rasselet@mines-ales.fr (D.R.); Jose-marie.lopez-cuesta@mines-ales.fr (J.-M.L.-C.)
[2] LMGC, IMT Mines Ales, University Montpellier, CNRS, 30319 Ales, France; monica.pucci@mines-ales.fr (M.P.); anne-sophie.caro-bretelle@mines-ales.fr (A.-S.C.-B.)
* Correspondence: aurelie.taguet@mines-ales.fr; Tel.: +33-(0)-466-785-687

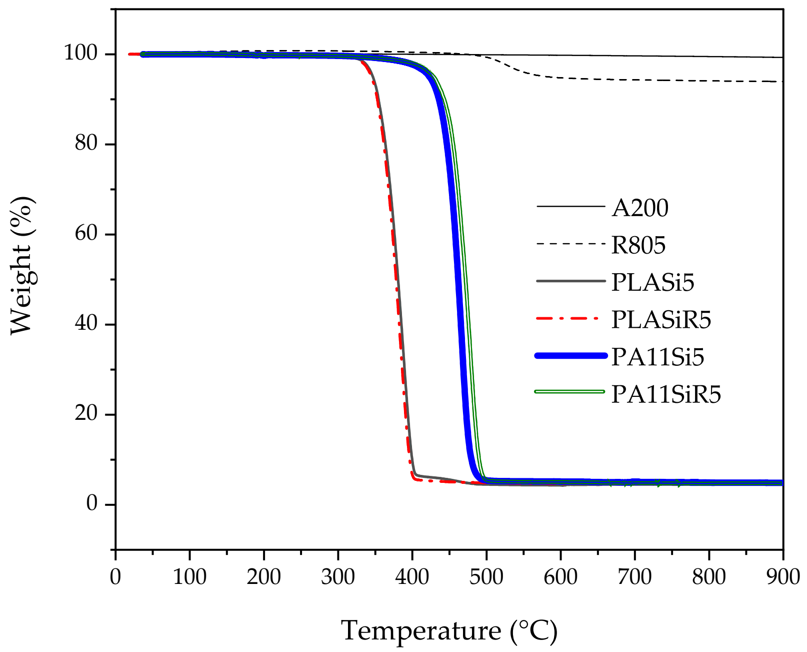

Figure S1. TGA measurements on pure silica (A200 and R805) and on neat polymer nanocomposites (PLA and PA11 with each silica A200 and R805). It must be noted that the DTG peak of neat PA11 is at 505°C whereas that of neat PLA is at 403°C.

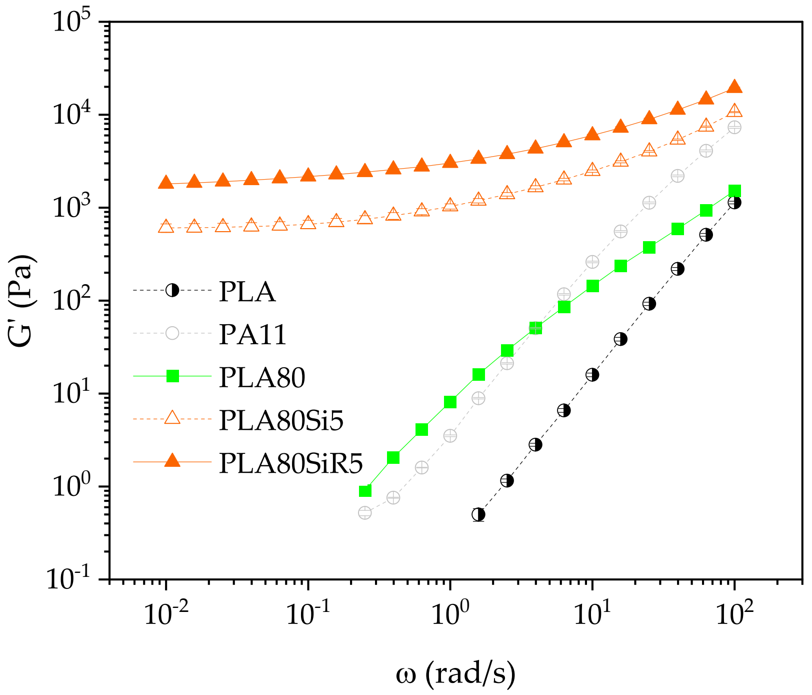

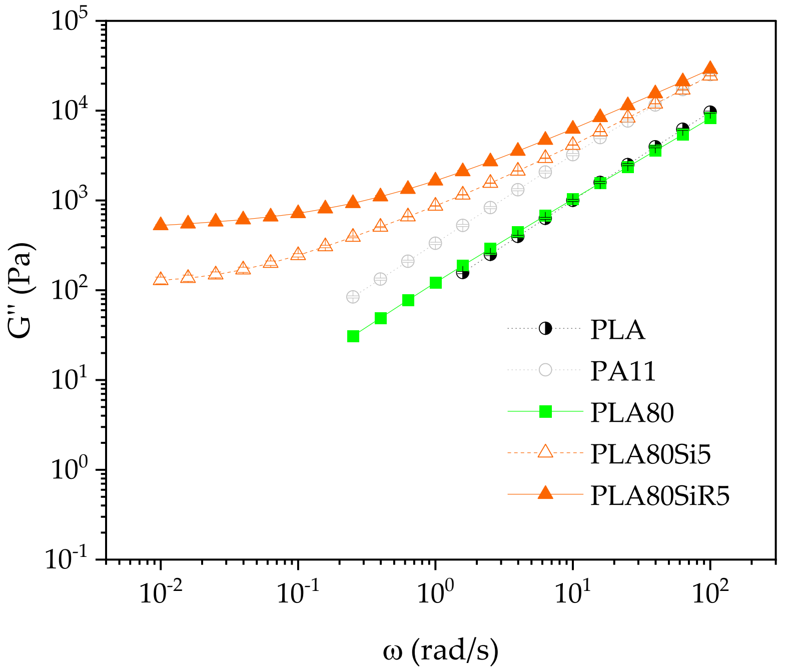

Figure S2. Frequency sweep tests giving the G′ & G′′ versus frequency for the neat polymers, PLA80 blend and both blend nanocomposites PLA80Si5 and PLA80SiR5.
